# Gut microbes from the phylogenetically diverse genus *Eubacterium* and their various contributions to gut health

**DOI:** 10.1080/19490976.2020.1802866

**Published:** 2020-08-23

**Authors:** Arghya Mukherjee, Cathy Lordan, R. Paul Ross, Paul D. Cotter

**Affiliations:** a Department of Food Biosciences, Teagasc Food Research Centre, Moorepark, Fermoy, Ireland; b School of Microbiology, University College Cork, Cork, Ireland; c APC Microbiome Ireland, University College Cork, Cork, Ireland

**Keywords:** *Eubacterium*, gut microbiota, short-chain fatty acids, *Eubacterium hallii*, *Eubacterium rectale*, butyrate, irritable bowel syndrome, phylogeny, bile acids, cholesterol

## Abstract

Over the last two decades our understanding of the gut microbiota and its contribution to health and disease has been transformed. Among a new ‘generation’ of potentially beneficial microbes to have been recognized are members of the genus *Eubacterium*, who form a part of the core human gut microbiome. The genus consists of phylogenetically, and quite frequently phenotypically, diverse species, making *Eubacterium* a taxonomically unique and challenging genus. Several members of the genus produce butyrate, which plays a critical role in energy homeostasis, colonic motility, immunomodulation and suppression of inflammation in the gut. *Eubacterium* spp. also carry out bile acid and cholesterol transformations in the gut, thereby contributing to their homeostasis. Gut dysbiosis and a consequently modified representation of *Eubacterium* spp. in the gut, have been linked with various human disease states. This review provides an overview of *Eubacterium* species from a phylogenetic perspective, describes how they alter with diet and age and summarizes its association with the human gut and various health conditions.

## Introduction

The importance of the gut microbiota in human health is now well established^1^. Components of the microbiota can facilitate the extraction of energy from nutrients, the deposition of fat in adipose tissues and provide for other resident microbes of the gut, besides eliminating pathogens through exclusion and other means.^2^ Depending on a plethora of factors that may be personal or environmental, the composition and function of the gut microbiota can vary significantly. However, distinguishing between a healthy or an unhealthy gut microbiome is difficult due to this large variability. Community composition alone, therefore, is not a reliable indicator of an aberrant or unhealthy state^1^ and, thus, a nuanced understanding of the microbiota, encompassing how specific taxa contribute to gut homeostasis and interact with their human host, is required for the development of evidence-based microbial therapeutics.^3,4^

Here, we focus on the genus *Eubacterium*, which was first proposed by Prévot in 1938 to describe a group of beneficial bacteria isolated from human feces.^5^ *Eubacterium* spp. are frequently encountered in the oral cavity and intestinal tract of mammals, including in the rumen of ruminants, as well as in the environment. The genus forms one of the core genera of the human gut microbiota and shows widespread colonization of the human gut across various human populations in Africa,^6,7^ Australia,^8^ Europe,^9^ India,^10^ South America,^11,12^ Asia^13^ and North America.^14,15^ Indeed, extensive human gut metagenome studies have reported the recovery of a large complement of metagenome-assembled *Eubacterium rectale* genomes irrespective of geographical location, age, lifestyle and clinical status.^16,17^ Interestingly, while *Eubacterium* spp. are routinely recovered from animal gut, an absence of *E. rectale* have been reported in primate gut; coupled with its omnipresence in the human gut this suggests a high degree of specificity and adaptation for the latter.^17^

Multiple species of the genus are currently regarded as promising targets for microbial therapeutics. Indeed, recent consensus among gut microbiologists suggests that specific strains of butyrate-producing microbes belonging to the genera *Eubacterium, Roseburia* and *Faecalibacterium*, among others, may ultimately be considered as beneficial to human health in the same manner as strains of *Lactobacillus* and *Bifidobacterium*.^18^ The genus *Eubacterium* is challenging to define, as discussed further below, and several species initially assigned to the genus have been subsequently reassigned to an existing or novel genus. Even now, the genus continues to be phylogenetically diverse and members can be assigned to several lineages. In recognition of this taxonomic flux, we will include some former *Eubacterium* species that have been recently reassigned to other genera for the purpose of this review. Additionally, we will largely restrict our discussion to *Eubacterium* species that are most relevant to the gut. Notably, even though much is known about the genus in general, our understanding of its function in the gut continues to evolve. Ultimately, here we review the literature to date relating to the phylogeny, characteristics and contributions of the members of the genus in relation to the human gut health and microbial ecology.

## The genus *Eubacterium* is phylogenetically diverse

The genus *Eubacterium* consists of Gram positive, uniform or pleomorphic non-spore forming, obligately anaerobic, and chemoorganotrophic bacterial rods. Species in this genus can be saccharoclastic or nonsaccharoclastic and motile or immotile in nature.^19^ Bacteria from this genus produce mixtures of organic acids from carbohydrates or peptone, which may include copious amounts of butyric, acetic and formic acids but do not produce: (a) only lactic acid, (b) propionic acid as the major acid, (c) greater quantities of acetic acid than lactic acid with or without the formation of formic acid and (d) lactic and succinic acid with small quantities of acetic or formic acid.^5^ This definition is rather loose and leads to the incorporation of species in the genus by default; historically resulting in the inclusion of species with a variety of phenotypes and genotypes in the genus and, ultimately, making it highly heterogeneous. According to the latest iteration of the Bergey’s Manual of Systematics of Bacteria and Archaea^19^ as well as NCBI Taxonomy, the genus *Eubacterium* belongs to the bacterial phylum *Firmicutes*, order *Clostridiales* and family *Eubacteriaceae*. However, according to the Genome Taxonomy Database (GTDB), which uses whole/draft genome information for classification of taxa, the genus should be assigned to the family *Lachnospiraceae*.^20^ The genus currently consists of 42–44 species depending on the taxonomy being followed, and the major species of interest in relation to the human gut include *Eubacterium rectale, E. hallii, E. ventriosum, E. eligens, E. coprostanoligens*, and *E. limosum*. The DNA G + C content (mol%) of the genus varies from 30 to 57% and the type strain is *Eubacterium limosum*.

**Figure 1.**
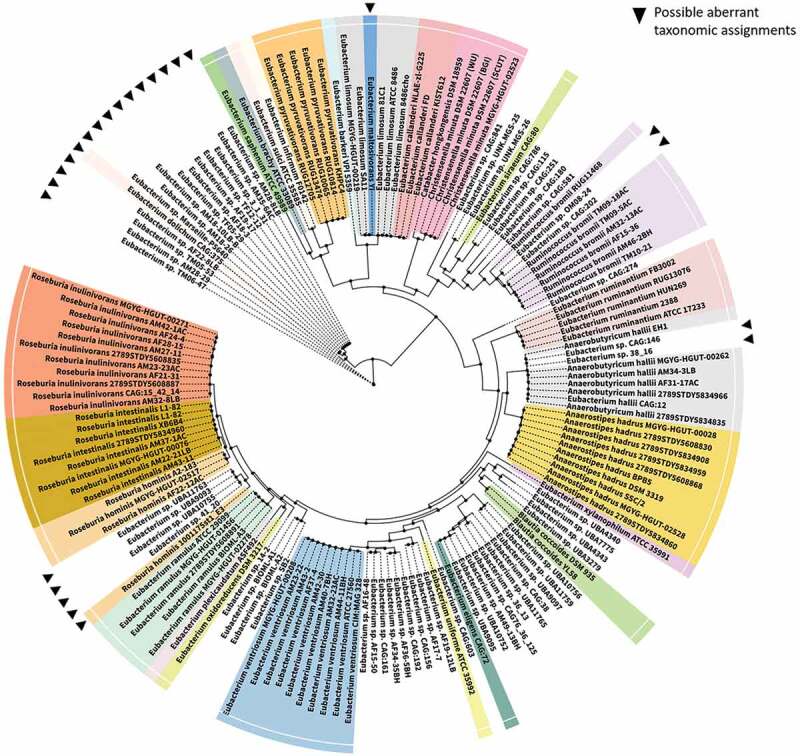
Phylogenetic relationship of *Eubacterium* spp. Complete genomes for *Eubacterium* species (current and recently reassigned) were obtained from NCBI along with other closely related gut microbes. 16 ribosomal marker proteins (including rpL14, rpL15, rpL16, rpL18, rpL22, rpL24, rpL2, rpL3, rpL4, rpL5, rpL6, rpS10, rpS17, rpS19, rpS3 and rpS8) were extracted from each genome, aligned with MAFFT v7.271^21^ and concatenated to create a RP16 protein alignment. Phylogenetic reconstruction using maximum likelihood was carried out in IQ-TREE^22^ with the following settings: -mset WAG,LG,JTT,Dayhoff -mrate E,I,G,I + G -mfreq FU -wbtl. Only genomes with at least 4 ribosomal marker proteins were included in the tree. The resulting tree was visualized using iTOL.^23^ Possible misclassifications are denoted by filled, inverted triangles in the phylogram. Tree nodes are depicted by filled circles.

Due to the rather loose definition of the genus, many of the species currently in the genus do not belong in the genus *sensu stricto* and are likely be moved to novel or existing genera in time. Indeed, 16S rRNA analysis of the species in genus *Eubacterium* has highlighted their wide distribution across phylogenetic trees.^5^ Here, we provide an update of this tree to display the phylogenetic relationship between some members of the genus and other closely related species using a representative maximum-likelihood tree constructed with 16 ribosomal protein markers (Figure 1). The majority of members of *Eubacterium* that have undergone taxonomic reassignment are assigned to phylum *Firmicutes* and are widely distributed therein. Examples include *E. formicigenerans* and *E. timidum*, which were reassigned to the genera *Dorea* and *Mogibacterium* respectively.^24,25^ However, several other members have been reassigned to other phyla; instances include the reassignment of *Eubacterium* species to genera such as *Slackia, Cryptobacterium* and *Eggerthela*, all of which belong to phylum *Actinobacteria*. Notably, certain *Eubacterium* species such as *E. cylindroides* may exhibit both Gram-positive and Gram-negative characteristics, thereby creating ambiguity in a fundamental phenotypic characteristic that is frequently implemented in taxonomic assignment; this contributes further to the considerable confusion in classification of *Eubacterium* species.^26^ It has been proposed that the core genotype of the genus *Eubacterium sensu stricto* be restricted to the type species of the genus, *Eubacterium limnosum*, along with *Eubacterium callanderi, Eubacterium barkeri* and *Eubacterium aggregans*,^19,27^ with the remaining species potentially assimilated into/reclassified as existing or novel genera when ample genomic and phylogenetic evidence supporting the same is available. For practicality, members have to date been grouped into subcategories based on phylogenetic characteristics. One of the loose phylogenetic subcategories proposed contain *E. rectale, E. oxidoreducens, E. ramulus, Roseburia cecicola* and *R. intestinalis*, where all species included except *E. oxidoreducens* produce butyrate and are saccharolytic.^19^ The taxonomic relationships of the members in this subcategory are discussed in detail by Duncan et al,^28^ and presents a strong case for reclassification of some of these species. *E. eligens*, an important gut *Eubacterium*, has been found to share considerable phylogenetic and phenotypic similarity with *Lachnospira pectinoschiza* and merits possible reclassification with availability of further evidence.^19^

Certain *Eubacterium* species that are important in relation to gut health have already undergone, or are proposed to undergo, reclassification in view of their divergent phenotypic and phylogenetic characteristics. For example, Shetty et al. proposed the reclassification of *E. hallii* as *Anaerobutyricum hallii* Comb. Nov., when reporting a similar novel butyrate and propionate-producing species *Anaerobutyricum soehngenii*.^29^ Indeed, *E. hallii*, along with *E. indolis, E. cellulosolvens, E. plexicaudatum, E. ruminantium, E. saburreum, E. xylanophilum, E. uniforme, and E. ventriosum* form a subcategory of interest in the genus *Eubacterium*. Notably, members of this group are not phylogenetically or phenotypically related to other species in the genus and exhibit distinct characteristics that warrants the creation of a novel genus for each.^19^ Another common intestinal inhabitant, *Eubacterium hadrum*, was also assigned to the genus *Anaerostipes*, based on both genotypic and biochemical features.^30^ Additionally, it has recently been proposed that one of the most important gut microbes, *E. rectale*, be reclassified as *Agathobacter rectalis*.^31^ This reclassification was however challenged by Sheridan et al.^32^ who argued that the evidence presented by Rosero et al.^31^ did not justify reclassification. The need for reclassification of *E. rectale* was however acknowledged by Sheridan et al., but urged exercising caution with this important member of the human gut, noting that any change in its taxonomic or phylogenetic affiliations will have a major impact on human microbiota research.

Understandably, taxonomic reassignments proposed have not been universally accepted yet and indeed, as noted, care must be taken while considering taxonomic classification and reporting of any member of genus *Eubacterium*. Further efforts relating to the classification of the genus should have a primarily genotypic focus with an emphasis on genomic characteristics. The prokaryotic taxonomy devised by Parks et al.^33^ in the GTDB, where a battery of universal, single copy marker genes derived from whole/draft genomes, are used to classify microorganisms, can be used as a model. Such an approach standardizes taxonomic assignments through normalization of taxonomic ranks on the basis of relative evolutionary divergence and has been shown to be capable of deconvoluting polyphyletic groups. Combined with rapidly declining sequencing prices, the increasing and ample availability of prokaryotic genomes can contribute greatly to such an exercise. With assembly of high-resolution draft genomes from metagenomes also now routine, microbiologists can glean information from truly uncultivable organisms and a definitive reclassification of the genus *Eubacterium* should be possible in the near future. Until then, there is likely to continue to be those who will view *Eubacterium* as a combined group – *Eubacterium et rel*. – when discussing human health, especially in relation to the gut. We will adopt this approach for the remainder of this review.

## Modulation of *Eubacterium* spp. in the gut by diet and age

Diet is one of the most important factors that dictates the composition and diversity of the gut microbiota. In case of *Eubacterium* spp., their presence in the gut have been largely associated with increased intake of dietary fibers and have been shown to decrease with an increasing protein/fat percent in diet.^34^ These observations are supported by recent studies outlining the utilization of digestion resistant complex carbohydrates by *Eubacterium* species.^35–37^ In a recent study by Scott et al., *E. hallii* and *E. rectale* were shown to be capable of utilizing media supplemented with resistant carbohydrates, i.e., fructans of increasing chain lengths such as P95 short-chain fructo-oligosaccharides, high-performance inulin, and Synergy-1; Dahlia inulin was metabolized exclusively by *E. rectale*.^35^ Several studies have reported that a Western diet, which includes increased proportions of animal protein and fat and is low in fiber, leads to a marked decrease in bacterial abundance in the gut including desirable taxa such as *Bifidobacterium* and *Eubacterium*.^38–40^ The corollary all appears to be true in that research involving the Mediterranean diet, which is well established as a diet that can contribute to health, has been shown to increase *Eubacterium* spp. populations in the gut.^41,42^ Other studies with diverging diets have also contributed to our understanding of how *Eubacterium* spp. is modulated in the gut. For example, Noriega et al. investigated changes in the gut microbiota when a polyunsaturated omega-3 fatty acid-rich diet was fed to a 45-year-old male.^43^ After the feeding phase, the fecal samples collected showed a drastic increase in abundances of several butyrate producers including *Eubacterium* spp. indicating a positive modulation of *Eubacterium* by polyunsaturated fatty acids. Further investigations, however, must be performed, to better understand the changes in *Eubacterium* spp. population in the gut with diet.

Through the process of aging, the gastrointestinal tract undergoes changes, including degeneration of the mucosal barrier and enteric nervous system along with an alteration of intestinal motility and an increase in gastrointestinal pathologies. As a general trend, microbiota diversity in the elderly is decreased with fewer butyrate producers and an increase in the number of potential pathogens.^44,45^ Among other things, a decrease in *s*hort-*c*hain *f*atty *a*cid (SCFA) production in the gut can result in an impaired secretion of mucins by the intestinal epithelial cells, providing enhanced access for pathogens to the intestinal mucosa and, potentially, gut inflammation.^46^ In elderly individuals, gut inflammation can be exacerbated by impairment of the gut-associated lymphoid tissue (GALT), leading to inefficient control of the resident microbiota and release of pro-inflammatory cytokines and chemokines by enterocytes; with the latter driving the differentiation of effector T_H_1, T_H_2 and T_H_17 cells.^47–49^ Consistent with these observations, a decrease in the relative proportion of *E. hallii, E. rectale*, and *E. ventriosum* has been noted in centenarians, whereas potentially pathogenic bacteria from the phylum *Proteobacteria* were increased.^50^ The beneficial effects of *Eubacterium* spp. were highlighted in an extensive study carried out by Ghosh et al., where a large cohort (n = 612) of elderly individuals were investigated to assess the modulatory effects of the Mediterranean diet on their gut microbiota.^42^ The authors reported that *Eubacterium* species such as *E. rectale* and *E. eligens* were positively associated with several markers of lower frailty and improved cognitive ability as well as increased short/branched chain fatty acid production. *Eubacterium* spp. also showed negative correlations with inflammatory markers such as IL-2 and C-reactive protein. Furthermore, network analysis revealed *Eubacterium* spp. to be a keystone species in the elderly gut microbial ecosystem, with frailty-associated taxa on the fringe. However, in contrast to these general observations, other studies have inferred a positive association between *Eubacterium* spp. and age.^51,52^ Clearly, inconsistent observations, albeit from studies involving diverse experimental designs with presumably quite variable diets, highlight the need for additional age-related studies in which other variables, especially diet, are as consistent as possible.

## Short-chain fatty acids produced by *Eubacterium* spp. contribute to gut health

**Figure 2.**
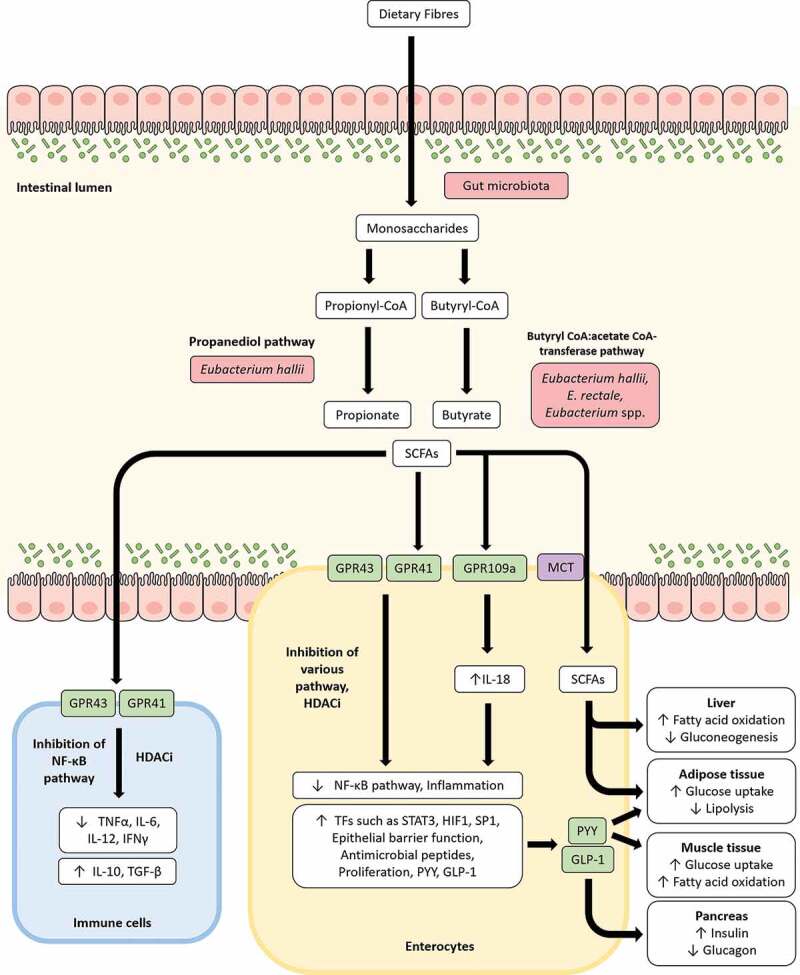
Modulation of various processes through short-chain fatty acids (SCFAs) produced by *Eubacterium* spp. Upon reaching the gut, carbohydrates resistant to digestion (commonly derived from dietary fibers) are degraded by gut microbiota to produce monosaccharides. These monosaccharides can be utilized by certain bacteria, including *Eubacterium* spp., in the gut to produce SCFAs such as butyrate, propionate, and acetate. SCFAs interact with G-protein-coupled receptors such as GPR43, GPR41, and GPR109a to modulate inflammation, intestinal barrier integrity, glycemic response, energy homeostasis and other host responses. Inflammation is suppressed by SCFAs primarily through inhibition of the NF-κB pathway and/or histone deacetylase function (HDACi) to downregulate pro-inflammatory cytokines such as TNFα, IL-6, IL-12, IFNγ and upregulate anti-inflammatory cytokines such as IL-10, TGF-β in a variety of cells including immune cells such as macrophages in lamina propria. IL-18 expression upregulated by GPR109a contributes to the enhancement of intestinal barrier integrity. SCFAs can also be taken up by enterocytes through the monocarboxylate transporter (MCT) and along with peptide YY (PYY) and glucagon-like peptide-1 (GLP-1) variably stimulates the liver, muscles, pancreas and adipose tissues to influence glycemic response, lipolysis, fatty acid oxidation and gluconeogenesis.

During the process of digestion, most available nutrients undergo absorption in the duodenum. However, a fraction of ingested carbohydrates that are resistant to digestion, including dietary fibers, remain intact until they reach the colon. Here, these *m*icrobiota *a*ccessible *c*arbohydrates (MACs) are fermented and metabolized by specialized enzymes produced by the resident gut microbiota^53^ (Figure 2). Microbial degradation of these complex carbohydrates, and of host mucin, results in the production of hexoses and pentoses before subsequent conversion to lactate and SCFAs such as acetate, propionate, butyrate, formate, and succinate by several other gut microbes.^54^ These SCFAs can, in turn, be taken up by the host. Unsurprisingly, ingestion of dietary fibers have been directly correlated to SCFA concentration and abundance of butyrate producers including *Eubacterium* spp., whereas high-fat diets have been associated with reduced formation of SCFAs.^34,55,56^ Additionally, the abundance of *Eubacterium* spp. in the gut is strongly correlated with SCFA levels and the beneficial effects of SCFAs in a variety of clinical conditions such as inflammatory bowel diseases, metabolic syndromes, and colorectal cancer, as discussed below.

### Production of butyrate and propionate by Eubacterium spp. in the gut

Among the SCFAs, propionate, and butyrate are most often considered to benefit human health and are produced by distinct cohorts of the colonic microbiota including several species from the genus *Eubacterium*^57^ (Figure 2). One of the most extensively studied *Eubacterium* species, *E. rectale*, was first isolated from the feces of healthy Japanese-Hawaiian males and identified as a major butyrate producer capable of utilizing complex carbohydrates such as cellobiose and starch for growth and proliferation.^58^ *E. hallii*, on the other hand, was first reported as a butyrate producer in the human gut by Barcenilla et al. in a 16S rRNA gene-based RFLP study.^59^ In the gut, butyrate can be produced from carbohydrates via glycolysis where two molecules of acetyl-CoA are combined to form acetoacetyl-CoA and undergo stepwise reduction to produce butyryl-CoA. Two distinct pathways are currently known for the final transformation of butyryl-CoA to butyrate; this proceeds either through the butyryl-CoA:acetate CoA-transferase pathway or the phosphotransbutyrylase and butyrate kinase pathway.^60^ The butyryl CoA:acetate CoA-transferase route for the final production of butyryl-CoA from acetyl-CoA is shared by *E. rectale* and the closely related *Roseburia* species, along with genomic organization of the butyrate synthetic genes. The same pathway is also employed by other *Eubacterium* species such as *E. hallii* and *E. biforme* (now reclassified as *Holdemanella biformis*) for the production of butyrate.^60^ Both *E. rectale* and *E. hallii* have been subsequently identified as prolific butyrate producers in the gut. Indeed, they were found to be among the 10 most abundant members of the human fecal microbiota, contributing more than 44% of butyryl-CoA:acetate CoA-transferase sequences retrieved from fecal samples of 10 healthy volunteers.^61,62^ A recent Swiss cohort study has also shown *E. hallii* to be one of the first producers of butyric acid in the infant gut.^63^

Propionate can be formed via two pathways from sugar fermentation by gut microbes. While the succinate pathway processes most pentose and hexose sugars to produce propionate, deoxy sugars such as fucose and rhamnose are metabolized by the propanediol pathway. The latter are readily available in the gut environment as dietary (e.g. human milk oligosaccharides) or host-derived (mucin) glycans and upon utilization by a variety of gut microbes can produce 1,2-propanediol as an end product. Although unable to degrade deoxy sugars, 1,2-propanediol can be metabolized by *E. hallii*, which has been shown to carry the glycerol/diol dehydratase PduCDE, a key enzyme in the transformation of 1,2-propanediol to produce propionate and propanol with the generation of one ATP.^64,65^ The conversion of 1,2-propanediol to propionate is dependent on the availability of vitamin B12 and occurs within microcompartments called polyhedral bodies to sequester the toxic intermediate, propionaldehyde.^66^

Although *Eubacterium* spp. can degrade complex carbohydrates, certain strains of *Eubacterium* spp. strains may lack the ability to degrade specific complex carbohydrates and rely on metabolites produced by other gut microbes for doing so; fermented products produced by these other gut microbes can then be utilized by *Eubacterium* spp.^57^ The importance of cross-feeding mechanisms in SCFA production by *Eubacterium* spp. have been demonstrated in a number of instances.^67–69^ In these studies, *Eubacterium* spp. were co-cultured with *Bifidobacterium* spp. in the presence of complex carbohydrates. The *Bifidobacterium* strains, which are capable of degrading complex carbohydrates such as arabinoxylan oligosaccharides and fucosyllactose, were shown to produce acetate, lactate and 1,2-propanediol, all of which were in turn taken up and used by *Eubacterium* spp. to produce butyrate and propionate. Evidence of such cross-feeding by *Eubacterium* spp. not only highlights the synergistic interactions between gut microbes and butyrogenic effects of resistant carbohydrates, but also underlines the ecological roles of *Eubacterium* spp. in the gut environment.

### Eubacterium spp. modulate gut inflammation through SCFAs

SCFAs, and especially butyrate has been reported to impart varied beneficial effects on human health.^70^ Although, the least abundant SCFA produced, butyrate constitutes the primary energy source of colonocytes, promoting their proliferation, maturation, and a healthy colon.^71^ Indeed, *E. rectale* has been reported to preferentially colonize the mucus layer, thereby increasing the bioavailability of butyrate for epithelial colonocytes.^72^ Additionally, SCFAs, have been shown to play a major role in modulation of inflammation in the gut through promotion of intestinal integrity and regulation of immune response. SCFAs can improve transepithelial resistance through upregulation of tight junction proteins such as claudin-1 and occludin as well as the intestinal mucin protein, mucin 2.^73,74^ Modification of various signaling pathways have been also described to highlight regulation of immune response by SCFAs including activation of G-protein-coupled receptors (GPCRs) and inhibition of histone deacetylases (HDACs).^75^ SCFAs can bind to at least four discrete GPCRs – FFAR2 (Free fatty acid receptor), FFAR3, GPR109a, and Olfr78 as ligands, albeit with varying specificity.^76^ For example, butyrate binds preferentially to FFAR3 over FFAR2, which exhibits higher affinities for acetate and propionate.^76^ FFAR2 is widely expressed in diverse tissues with highest expression in immune cells. Several studies have shown that SCFAs can act as an anti-inflammatory agent through inhibition of pro-inflammatory cytokines such as IFN-γ, IL-1β, IL-6, IL-8, and TNF-α, while upregulating anti-inflammatory cytokines such as IL-10 and TGF-β in a FFAR2/FFAR3 dependent manner.^75,77^ GPR109a activates the inflammasome pathway in colonic macrophages and dendritic cells, thereby inducing differentiation of regulatory T cells, and anti-inflammatory IL-10 producing T-cells.^78^ GPR109a activation by SCFAs in intestinal epithelial cells (IECs) can also increase production of IL-18, a key cytokine for repair and maintenance of intestinal epithelial integrity.^79^ Inhibition of HDAC activity by propionate and butyrate have been associated with the downregulation of expression for pro-inflammatory cytokines and chemokines such as CXCL8 and CCL20 in IECs.^80^ HDAC inhibition by SCFAs have also been associated with the increase in expression of β-defensins and cathelicidins such as LL-37.^81^ Given the extensive involvement of SCFAs in modulation of gut health as described, especially butyrate, a dysbiosis of the gut microbiota involving SCFA producers has major implications due to alteration of the SCFA profile in the intestine.^82^

Inflammatory bowel diseases (IBDs) are severe and chronic inflammations of the gastrointestinal tract and are characterized by two major clinical phenotypes: Crohn’s disease (CD) and ulcerative colitis (UC). CD involves the transmural inflammation of all layers of the epithelial wall, whereas UC only affects the superficial mucosal layer. In general, IBDs recurrently exhibit dysbiosis of the gut microbiota that is characterized by a decrease in the diversity and temporal stability of the microbiota. While the exact role of microbial disturbances in the pathogenesis or causation of IBDs is still being elucidated, the proportion of butyrate producers including *Eubacterium* spp. in the gut are consistently reduced in IBD subjects^83-85^ (Table 1). Indeed, a decreased abundance of clostridial clusters IV and XIVa in IBD patients compared to non-IBD, healthy individuals along with a concomitant increase in proteobacterial pathobionts constitute a signature for microbial dysbiosis in IBDs and can be considered as biomarkers.^86,95-97^ Consequently, a decrease in gut butyrate levels is commonly observed in patients suffering from IBD, leading to improper modulation of the host immune system.^98^ Decreased levels of SCFA in the gut in IBD and experimental colitis have also been correlated with reduced regulatory T cell functionality and increased inflammation.^99,100^

**Table 1.** Case-control studies showing association of *Eubacterium* spp. with inflammatory bowel diseases (IBDs).

Pathology/condition/cohort description	Principal method(s) used	Inferences	Reference
Fecal samples from 6 children with Crohn’s disease (CD), 6 children with ulcerative colitis (UC) and 12 healthy siblings.	Metagenomic shotgun sequencing	↓ *E. rectale* and *F. prausnitzii* *↑ E. coli* and *Fusobacterium nucleatum* in IBD patients.	Knoll et al.^86^
Fecal samples were obtained from 6 healthy volunteers (median age of 26.5) and 6 UC patients (median age of 40.5). Healthy volunteers: 5 males and 1 female; UC group: 3 males and 3 females.	M-SHIME system, Denaturing Gradient Gel Electrophoresis (DGGE), qPCR	↓ *E. rectale* and *C. coccoides*. Reduced luminal butyrate in M-SHIME samples inoculated with feces from UC patients.	Vermeiren et al.^87^
35 Saudi children with (n = 17) or without (n = 18) CD. The median age was 15 years for children with CD and 16.3 years for healthy controls. Gender distribution indicated that 65% of the CD patientand 67% of the control subjects were males.	16S rRNA gene sequencing	*Roseburia inulivorans* ↓, *Eubacterium seraeum* ↓, *Eubacterium* spp. ↓ Several species including the ones shown above were depleted in children with CD.	El Mouzan et al.^85^
Fecal samples were collected from pediatric patients (CD: n = 10 and UC: n = 12) along with healthy children (n = 8).	Polyphasic microbiological analysis including culture-based study, real-time PCR, and DGGE	↓ *Bifidobacterium sp*. and *E. rectale* in both UC and CD patients.	Maukonen et al.^88^
Mucosal biopsies of both inflamed and non-inflamed sites from 14 patients with active UC. Paired mucosal biopsies of the corresponding sites obtained from 14 non-IBD controls.	16S rRNA gene sequencing	Significantly decreased microbial diversity in both inflamed and non-inflamed sites in UC patients compared with non-IBD controls. Decreased abundance of the genera *Prevotella, Eubacterium, Neisseria, Leptotrichia, Bilophila, Desulfovibrio, Butyricimonas* at inflamed site of UC patients.	Hirano et al.^89^
Fecal and blood samples from 68 pediatric patients with IBD (males = 38) and 26 controls (males = 11). 32 patients received anti-tumor necrosis factor-α (anti-TNF-α).	Phylogenetic microarray, qPCR	↓ microbial richness, abundance of butyrate producers, and relative abundance of *Clostridium* clusters IV and XIVa. Higher levels of baseline *E. rectale* and *Bifidobacterium* spp. predictive of successful response to anti-TNF-α medication.	Kolho et al.^90^
The mucosa associated colonic microflora of 57 patients (CD: n = 26; UC: n = 31) with active IBD and 46 controls were investigated.	16S rDNA based single strand conformation polymorphism (SSCP) fingerprint, cloning experiments, and real time polymerase chain reaction (PCR).	Decrease in microbial diversity in IBD. ↓ *Bacteroides* sp., *Eubacterium* sp., and *Lactobacillus* sp. in IBD patients compared to healthy subjects.	Ott et al.^91^
Fecal samples from 6 CD patients and 6 healthy volunteers.	Custom phylogenetic array, qPCR	In controls: ↑ *E. rectale, Bacteroides fragilis* group, *B. vulgatus, Ruminococcus albus, R. callidus, R. bromii*, and *F. prausnitzii*. In CD patients: ↑ *Enterococcus sp., Clostridium difficile, Escherichia coli, Shigella* *flexneri*, and *Listeria* sp.	Kang et al.^84^
104 *de novo* IBD-patients (63 CD, 41 UC, median age 14.0 years) and 61 healthy controls (median age 7.8 years).	IS-Pro assay	*Eubacterium* spp. were less abundant in IBD patients compared to healthy controls.	Meij et al.^92^
Fecal samples from 13 new-onset CD patients (9 females, 67%; mean age 32.18) and 16 healthy controls, matched by age and gender.	16S rRNA gene sequencing	↓ *Ruminococus, Roseburia, Parabacteroides, Mesoplasma, Faecalibacterium, Eubacterium* and *Alistipes* in CD samples compared to healthy controls.	Rojas-Feria et al.^93^
447 children and adolescents (<17 years) with new-onset CD and a control population of 221 subjects were enrolled as part of the RISK cohort. Biopsies were collected from the terminal ileum and rectum.	16S rRNA gene sequencing, metagenomic shotgun sequencing	In CD samples compared to healthy controls: ↑ *Escherichia coli, F*. *nucleatum, Haemophilus parainfluenzae*. ↓ *Bifidobacterium* *bifidum, B. longum, B. adolescentis*, *B. dentum, Blautia hansenii, F. prausnitzii, Ruminoccus* *torques, Clostridium bolteae, E. rectale*, *Roseburia intestinalis, and Coprococcus comes*.	Gevers et al.^94^

A butyrate-mediated protective effect provided by *Eubacterium* spp. in IBDs has been demonstrated in several recent studies. *In vitro* studies using fecal microbiota from UC and CD patients, represented by fewer butyrate producers, exhibited a decreased capacity for colonization and butyrate production; supplementation of the IBD microbiota with known butyrate producers including *Eubacterium* spp. restored butyrate production and improved epithelial barrier integrity and colonization capacity.^87,101^ The role of the *Eubacterium* spp.-butyrate-anti-inflammation axis in gut health was further demonstrated in children suffering from IBD who underwent an anti-TNF-α treatment; patients harboring a higher baseline abundance of *E. rectale* were more responsive to treatment with the presence of *E. rectale* being predictive of successful attenuation of inflammation.^90^ The protective effects of *E. limosum*, and the SCFAs it produces, in gut inflammation have been demonstrated in *in vitro* and murine models.^102^ SCFAs produced by *E. limosum* induced T84 colonocyte growth and reduced IL-6 and TLR4 expression by the colonocytes when stimulated by TNF-α treatment, with butyrate being the most prominent effector. Additionally, when provided with a 5% *E. limosum* chow, mice showed significant retention of body weight and colon length compared to the control group upon induction of colitis. These observations exhibit a butyrate-mediated anti-inflammatory effect of *Eubacterium* spp. on gut health and presents it as an attractive biotherapeutic in inflammatory gut ailments.

### Effects of SCFA production by Eubacterium spp. in Type II diabetes mellitus (T2DM) and obesity

The association of *Eubacterium* spp. with obesity remains controversial so far, with several reports suggesting a positive correlation of *Eubacterium* spp. with obesity.^103–105^ BMI is often considered a proxy for adiposity; some BMI-based studies have also reported greater abundances of *Eubacterium* spp. in obese subjects.^106,107^ Interestingly, higher levels of total butyrate have been reported in obese individuals with reduced fecal SCFAs in treated obese subjects, which suggests an enhanced assimilation of carbohydrates and lipids that can contribute to the obese phenotype.^108–110^ Such an observation can explain the higher abundance of butyrate producers including *Eubacterium* spp. in obese individuals. A closer look at dietary intervention studies indicates that the proportions of *Eubacterium* spp. and other butyrate producers in obese subjects may be influenced primarily by diet. A significant reduction of *Eubacterium* spp. is consistently reported in several studies where availability of complex carbohydrates to gut microorganisms have been restricted in obese individuals.^15,111,112^ A study by Balamurugan et al. in obese and non-obese Indian children exposed to similar diets also did not find any difference in *E. rectale* abundance between groups.^113^ Taken together, current evidence indicates that *Eubacterium* spp. along with other butyrate producers, when maintained in the gut through a consistent availability of reasonable amount of complex carbohydrates, increases in obese individuals in proportion, thereby facilitating energy extraction in the gut. It is also possible that diet rather than altered metabolic parameters in obese individuals, may drive the growth and proliferation of butyrate producers including *Eubacterium* spp. The exact mechanisms through which the gut microbiota may modulate obesity are still being elucidated. Instances where butyrate have been shown to alleviate diet-induced obesity and improve glucose homeostasis make it difficult to make linear conclusions and provides an incentive for further investigations.^114,115^ Ultimately, care must be taken while inferring direct associations between taxa and obesity, as such conclusions may be misleadingly oversimplistic for a metabolic syndrome with multifactorial influences.

*Eubacterium* spp. and butyrate producers have been positively associated with insulin sensitivity in several studies.^116,117^ Recent independent studies which compared metagenomes from healthy and T2D individuals, have clearly indicated a potential correlation between gut microbiota and T2D pathophysiology.^118,119^ The studies, carried out in Chinese and European populations, both reported a significant reduction of butyrate producers including *Eubacterium* spp. in T2D subjects.^109,119^ Additional studies have demonstrated the restorative effect of butyrate producers, including *Eubacterium* spp., transplanted from lean individuals, in both human and murine insulin-resistant models.^120–122^ Indeed, the increase in *Eubacterium* spp. after FMT was associated with metabolic improvement in insulin-resistant individuals.^120^ When orally administered to obese and insulin-resistant *db/db* mice, *E. hallii* have been shown to significantly improve insulin sensitivity and energy metabolism.^121^ The stimulation of gut hormones and inhibition of food intake by SCFAs have been proposed as possible mechanisms of modulation of host metabolism by gut microbiota in T2D individuals.^123^ Such a proposed mechanism is consistent with the observation that butyrate and propionate bound to FFAR2 receptor can regulate satiety hormones such as ghrelin (orexigenic peptide), glucagon-like peptide-1 (GLP-1), and peptide YY (PYY) (anorexigenic peptide)^124^ (Figure 2). Ghrelin, also known as the ‘hunger hormone’, stimulates appetite and is secreted before a meal, while GLP-1 and PYY are synthesized and released by enteroendocrine L cells and stimulate insulin secretion by pancreatic β cells, reduces food intake, and normalizes energy intake and weight loss. An opposite regulation of ghrelin and GLP-1/PYY by SCFAs, where GLP_1/PYY are upregulated and ghrelin is downregulated, ensures reduced food intake, satiety and reduced adiposity.^125^ Ghrelin, has also been negatively associated with the butyrate-producing *E. rectale*.^126^ Recent evidence from Zeevi et al., who performed a machine learning-based study on a large cohort (*n* = 800) in order to predict personalized postprandial glycemic response for individuals using an integrated feature dataset derived from dietary habits, gut microbiota, anthropometrics, physical activity, and blood parameters, also supports an affirmative role of *Eubacterium* spp. in insulin sensitivity.^127^ In their study, 72 features from the gut microbiome were inferred to be predictive, among which *E. rectale* was reported to be one of the most robust with a higher abundance of the bacterium in the gut being positively associated with lower postprandial glycemic response (*n* = 430). Butyrate produced by *Eubacterium* spp. can also provide additional benefits to T2DM patients through HDAC inhibition-mediated pancreatic β-cell reprogramming to improve insulin sensitivity and satiety.^128^ Finally, low-grade inflammation has been reported in T2DM, where inflammatory molecules are upregulated in insulin target tissues and contribute to insulin resistance.^129^ For example, TLR4-dependent increase in production of pro-inflammatory cytokines through activation of macrophages and β-cells in pancreatic islets leads to dysregulation, functional impairment, and decreased viability of β-cells.^130^ SCFAs produced by *Eubacterium* spp. can contribute to restoration of physiological inflammatory environments through mechanisms detailed above. Such a connection is also reinforced by the consistent decrease of other gut butyrate producers in T2DM.^116,117^ Current observations therefore consistently indicate *Eubacterium* spp. as a positive contributor in alleviating T2DM and should be considered as a potential therapeutic.

### Butyrate-mediated contribution of Eubacterium spp. in inhibition of colorectal cancer and atherosclerosis

Dysbiosis of the gut microbiota is closely associated with incidence of various cancers including colorectal cancer. While chronic inflammation and reduced immune response resulting from dysbiosis has been reported to contribute to increased cancer incidence, commensal bacteria have been demonstrated to increase immune surveillance and decrease cancer incidence.^131,132^ Dietary fibers have been associated with lower risks of intestinal cancer development; this is primarily due to the strong anti-cancer effect of butyrate.^133–136^ Through modulation of various signaling pathways involved in cell survival and apoptosis, the anti-cancer activity of butyrate has been demonstrated in cancer cells and mouse models.^137,138^ Butyrate, while being the preferred energy source for colonocytes, is poorly metabolized in cancer cells due to the Warburg effect. This leads to cytoplasmic accumulation and subsequent translocation of butyrate into the nucleus where it acts as a HDAC inhibitor and negatively modulates PI3K/Akt and JAK2/STAT signaling pathways, resulting in inhibition of carcinogenesis and increased cancer cell apoptosis.^138^ Additionally, inhibition of signaling pathways such as NF-κB and HIF-1 by butyrate have been reported to increase anti-cancer immune responses.^139^ Interestingly, the anti-carcinogenic effect of butyrate is dose-dependent; a lower concentration of the butyrate (0.5–1 mM) promotes growth of non-cancerous colonocytes and apoptosis in cancerous ones, whereas at higher concentrations (greater than 2 mM) it can cause apoptosis in both.^140,141^ Indeed, butyrate induces proliferation of colonocytes at the cript of the colon, where its concentration is lower, but shows a pro-apoptotic effect closer to the lumen where its concentration increases; this also ensures normal turnover of cells in the intestine.^142^

Butyrate producers including *Eubacterium* spp. are decreased in abundance in patients suffering from CRC.^143^ Indeed, gut microbiomes in CRC patients are less fermentative in nature with significantly decreased abundance of butyrate fermenters from *Clostridium* cluster XIVa such as *Eubacterium* sp. and *Roseburia* sp.; depletion of the butyryl-CoA transferase in CRC subjects have also been reported.^136,143^ Among *Eubacterium* spp., *E. rectale, E. hallii* and *E. ventriosum* are reported to be significantly reduced in abundance in the gut of individuals with CRC^136,143-145^ (Table 2). *E. ventriosum* has been proposed as biomarker for low risk of CRC, with significant enrichment in healthy individuals compared to CRC patients in diverse populations.^145^ Additionally, butyrate levels in the colon share an inverse relationship with the incidence of CRC.^148,149^ This can be attributed to gut dysbiosis in patients with CRC, where butyrate-producing capacity of the gut microbiota is significantly reduced.^150,151^ Such change in gut microbiota can be caused by a consistently lower intake of dietary fibers and consequent-decreased levels of SCFAs, as often observed in individuals with CRC.^136^ In the absence of butyrate, the intestinal tract can reach a state of chronic inflammation, that contributes to development and progression of CRC.^152,153^ Such consistent observations suggest there is merit in investigating the use of *Eubacterium* spp. strains as therapeutic interventions in CRC and related diseases. Indeed, Feng et al. have been granted patent rights for the use various strains of *E. ventriosum* and *E. eligens* in treating colitis and/or CRC.^154^

**Table 2.** Case-control studies showing association of *Eubacterium* spp. with colorectal cancer and atherosclerosis.

Pathology/condition/cohort description	Principal method(s) used	Inferences	Reference
Colorectal cancer (CRC)			
46 CRC patients, aged 42–77 years and 56 healthy volunteers, aged 40–54 years.	16S rRNA pyrosequencing of the V3 hypervariable region, real-time qPCR	Following trends were observed in CRC patients compared to healthy volunteers: ↓ *Bacteroides, Roseburia, Alistipes, Eubacterium, Parasutterella* ↑ *Porphyromonas, Escherichia/Shigella, Enterococcus, Streptococcus, Peptostreptococcus*	Wang et al.^143^
Gut metagenome datasets from two European studies with a combined 124 healthy control subjects and 99 CRC patients.	*In silico* study using zero-inflated lognormal models for estimation of relative abundance; diversity and network analysis.	Compared to healthy volunteers, *Eubacterium hallii, Anaerostipes hadrus, and Eubacterium ventriosum, Flavonifractor, Catenibacterium* and *Gardnerella* were found to be significantly decreased in CRC patients.	Ai et al.^144^
Three cohorts from China and Denmark with cohort C1 comprised of 128 individuals: 74 patients with CRC and 54 controls; cohort C2 comprised of 156 individuals: 47 patients with CRC and 109 controls. Cohort D comprised of 40 individuals including 16 patients with CRC and 24 control subjects.	Metagenomic shotgun sequencing, qPCR	Cross ethnic examination of metagenomes from CRC individuals identified *Eubacterium ventriosum* as a biomarker significantly associated against CRC, while *Parvimonas micra, Solobacterium moorei* and *F. nucleatum* shown to be consistently associated with it.	Yu et al.^145^
344 Chinese individuals each in healthy control group and advanced colorectal adenoma group	V1-V3 16S rRNA pyrosequencing, qPCR	Genera related to the fermentation of butyrate (*Clostridium, Roseburia*, and *Eubacterium* spp.), were significantly lower in the colorectal adenoma group compared to healthy control subjects, whereas the prevalence of *Enterococcus, Streptococcus*, and *Bacteroidetes* spp. was significantly higher.	Chen et al.^136^
Atherosclerosis/Atherosclerotic cardiovascular disease			
218 individuals with atherosclerotic cardiovascular disease (ACVD) and 187 healthy control subjects.	Metagenomic shotgun sequencing, Network analysis	Butyrate producing bacteria such as *Eubacterium* spp. *F. prausnitzii* and *Clostridiales* sp. were found to be significantly depleted in ACVD patients.	Jie et al.^146^
12 patients with symptomatic atherosclerotic plaques (who had undergone carotid endarterectomy for minor ischemic stroke, transient ischemic attack or amaurosis fugax) and 13 control subjects without large vulnerable plaques in the carotid arteries.	Metagenomic shotgun sequencing	Butyrate producers *Eubacterium* and *Roseburia* were found to be enriched in control subjects while atherosclerotic individuals were enriched in *Colinsella*.	Karlsson et al.^147^

Recent studies in humans and mice show that butyrate producers in the gut including *Eubacterium* spp. are negatively associated with atherosclerotic cardiovascular disease (ACVD).^146,147,155^ Deep analysis of gut metagenomes from atherosclerotic subjects show a depletion of butyrate producers such as *Eubacterium* spp., *Roseburia* spp. and *F. prausnitzii*, compared to healthy individuals^146,147^ (Table 2). The gut environment in ACVD subjects have consequently been reported to be less fermentative and inflammatory in nature.^146^ Notably, peptidoglycan (PG) and lipopolysaccharide (LPS) are pro-inflammatory, microbial pathogen-associated molecular patterns (PAMPs) that are recognized as risk factors in cardiovascular diseases (CVDs).^156^ PG biosynthesis genes are enriched in ACVD metagenomes, which indicates greater peptidoglycan production that can lead to priming of the innate immune system and inflammation.^147^ PG has also been observed in atherosclerotic plaques, while patients with a high CVD burden exhibit greater risk from circulating endotoxemia.^156^ Interestingly, network analysis of ACVD gut microbiomes have revealed that microbes enriched in ACVD have a mutually exclusive relationship with butyrate producers including *Eubacterium* spp., thereby suggesting consistency of dysbiosis in ACVD patients that is represented by a depletion of butyrate producers.^146^ Indeed, butyrate producers have been proposed to be protective against atherosclerosis.^155^ In atherosclerotic mice, negative association of *Eubacterium* spp. and other butyrate producers with plasma cholesterol, MMP-9 and A-FABP (biomarkers for cardiovascular pathologies) have been reported.^155^ It currently remains unclear if a dysbiotic gut is a read-out of atherosclerotic symptoms or vice-versa. However, since ACVD are manifestations of several factors such as lifestyle, diet and genetics, it is possible that lifestyle and diet may primarily contribute to a dysbiotic microbiota, which in turn may aggravate atherosclerotic development. Depletion of butyrate producers including *Eubacterium* spp. in the gut leads to reduced barrier integrity and translocation of pro-inflammatory microbial components such as PG and LPS across the intestinal epithelium. Inflammatory responses triggered by TLR4 bound by circulating LPS, primarily through the NF-κB pathway and the subsequent release of pro-inflammatory cytokines such as TNF-α, IL-6, IL-1, and IL-27, promotes the development of atherosclerosis.^156^ Additionally, nucleotide-binding oligomerization domain-containing protein 1 (NOD1) and NOD2 can bind circulating PG and activate the NF-κB pathway to trigger inflammatory responses. Indeed, NOD1/2 knockouts in murine models have revealed these receptors as critical in maintaining intestinal barrier integrity and development of atherosclerosis.^156^ Butyrate producers including *Eubacterium* spp., therefore, may play an important role in the gut-heart axis; they can restore a dysbiotic gut microbiota and modulate inflammation in ACVD subjects and merit further exploration as potential therapeutics.

## Transformation of cholesterol by *Eubacterium* spp. provides protection against cardiovascular diseases

**Figure 3.**
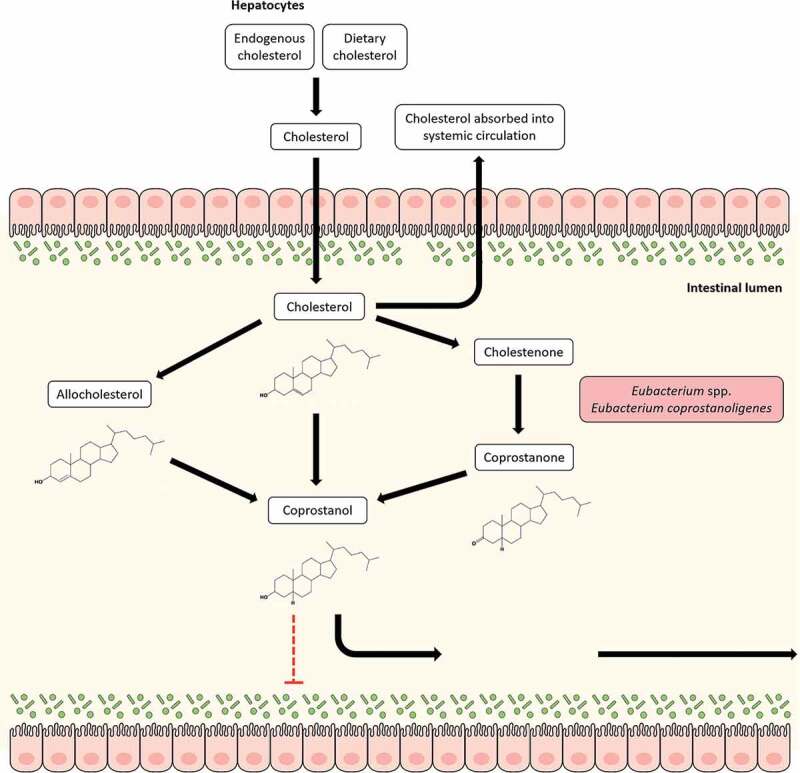
Cholesterol metabolism by *Eubacterium coprostanoligenes* in the gut. Cholesterol can reach the gut from two sources: endogenous (synthesized in the liver) or exogenous (in the form of dietary uptake). Cholesterol can be reabsorbed from the gut. The cholesterol that is not reabsorbed can be metabolized by *Eubacterium coprostanoligenes* to coprostanol both directly and indirectly through the intermediate, coprostanone. It can also reduce cholesterol to coprostanol upon epimerization to allocholesterol through a pathway that remains poorly studied. Unlike cholesterol, coprostanol is taken up poorly in the intestine and most of it is excreted in feces, thereby providing a route for cholesterol removal from the gut and systemic circulation.

Conversion of cholesterol to coprostanol by intestinal bacteria was first reported in the 1930s and several studies have been carried out since to identify bacteria capable of transforming cholesterol to coprostanol. Many of the identified microbes were eventually assigned to the genus *Eubacterium; Eubacterium coprostanoligenes* HL (ATCC 51222) represents one such bacteria that was isolated from a hog sewage lagoon and has received considerable attention due to its cholesterol-reducing properties.^157^ Although subsequently *Bacteroides dorei, Lactobacillus* sp. and *Bifidobacterium* sp. have been reported to have cholesterol utilization properties, these seem to be transient properties and may be lost, making *E. coprostanoligenes* HL the only available culturable gut isolate able to degrade cholesterol.^158^ The presence of *E. coprostanoligenes* in the gut microbiota has been strongly associated with fecal coprostanol.^158^ Recently, 3β-hydroxysteroid dehydrogenase homologs of *E. coprostanoligenes* have been identified in gut metagenomes that can transform cholesterol to coprostanol.^158^ Interestingly, these intestinal sterol metabolism A genes (*ismA*) have been attributed to yet uncultured gut microbes which formed a coherent clade with *E. coprostanoligenes* in the tree of life and may represent novel *Eubacterium* species involved in cholesterol reduction in the gut.^158^ The mechanism of cholesterol to coprostanol conversion has been investigated with three major proposed pathways and *Eubacterium* spp. have been found to be involved in all of these (Figure 3). The first pathway involves a direct, stereospecific reduction of the 5,6-double bond in cholesterol,^159^ while the second is an indirect pathway which includes at least three steps. The latter pathway, which has been demonstrated in *E. coprostanoligenes* HL, requires NADP^+^ and proceeds through the production of cholestenone and coprostanone intermediates.^158–160^ An additional third pathway has also been identified which involves isomerization of cholesterol to allocholesterol, which can be reduced to coprostanol by *Eubacterium* ATCC21-403 and 408 species.^161^ The final pathway is, however, poorly studied.

Nearly one gram of cholesterol from dietary and extra-dietary sources reach the human colon daily, where it is metabolized by commensal gut bacteria to coprostanol. Unlike cholesterol, coprostanol is poorly absorbed in the intestine, and is suggested to have an impact on modulation of cholesterol metabolism and serum cholesterol levels.^162^ This notion has been reinforced by findings that an inverse relationship exists between plasma cholesterol levels and the ratio of cholesterol to coprostanol in the feces.^163^ Cholesterol conversion to coprostanol has been therefore considered as a new strategy for management of cholesterol homeostasis in humans. As an extension, *Eubacterium* spp., which are highly involved in coprostanol metabolism in the gut have been investigated for their hypocholesterolemic effects. Li et al. reported a reduction in the plasma cholesterol levels and an increase in the coprostanol/cholesterol ratios in the digestive contents of hypercholesterolemic rabbits that were fed *E. coprostanoligenes*.^164^ The effects observed in these rabbits were further ascribed to cholesterol reduction by *E. coprostanoligenes* due to its preferential colonization in the jejunum and ileum, both of which are sites for cholesterol absorption. Similar observations have also been reported in germ-free mice.^165^ Additional results from a combined metabolomic and metagenomic study have identified multiple bacterial phylotypes including *Eubacterium eligens* ATCC 27750 (*p* = 1.477e-02) to be significantly correlated to high fecal coprostanol.^166^

Atherosclerotic cardiovascular diseases (CVDs) are widely recognized as a major public health concern, where key risk factors in their development include an imbalance in blood cholesterol levels and high serum concentrations of low-density lipoprotein cholesterol.^167^ Indeed, patients with ACVDs have higher cholesterol absorption in the gut.^158,168^ Notably, changes in the gut microbial community have been directly correlated to the rate of cholesterol converted to coprostanol, while a high efficiency of cholesterol transformation to coprostanol has been linked to a reduced risk of CVDs.^158,169,170^ Due to their hypocholesterolemic effect, *Eubacterium* spp. and other cholesterol-reducing microbes can provide protection against CVDs. Indeed, gut *Eubacterium* spp. in atherosclerotic subjects show a significantly negative correlation with established markers of atherosclerosis such as low-density lipoproteins, cholesterol and white blood cells.^147^ *E. coprostanoligenes* were also reduced in the murine gut when mice were fed a methionine-choline diet to induce nonalcoholic steatohepatitis, where damage to the liver inhibits the production of endogenous cholesterol.^171^ Furthermore, cholesterol-reducing homologs of *E. coprostanoligenes* 3β-hydroxysteroid dehydrogenase identified in metagenome-assembled genomes from gut metagenomes have been associated with lower levels of serum cholesterol; the *ismA* genes were also correlated to higher levels of coprostanol and lower levels of cholesterol in stool.^158^ The reduction of cholesterol to coprostanol still remains poorly understood and only few studies on cholesterol metabolizing bacteria are currently available. Greater investigative investment is necessary to garner a holistic understanding of the molecular mechanisms behind cholesterol-coprostanol metabolism in the gut, including *Eubacterium* spp., and to perfect hypocholesterolemic strategies.

## *Eubacterium* spp. contribute to gut and hepatic health through modulation of bile acid metabolism

**Figure 4.**
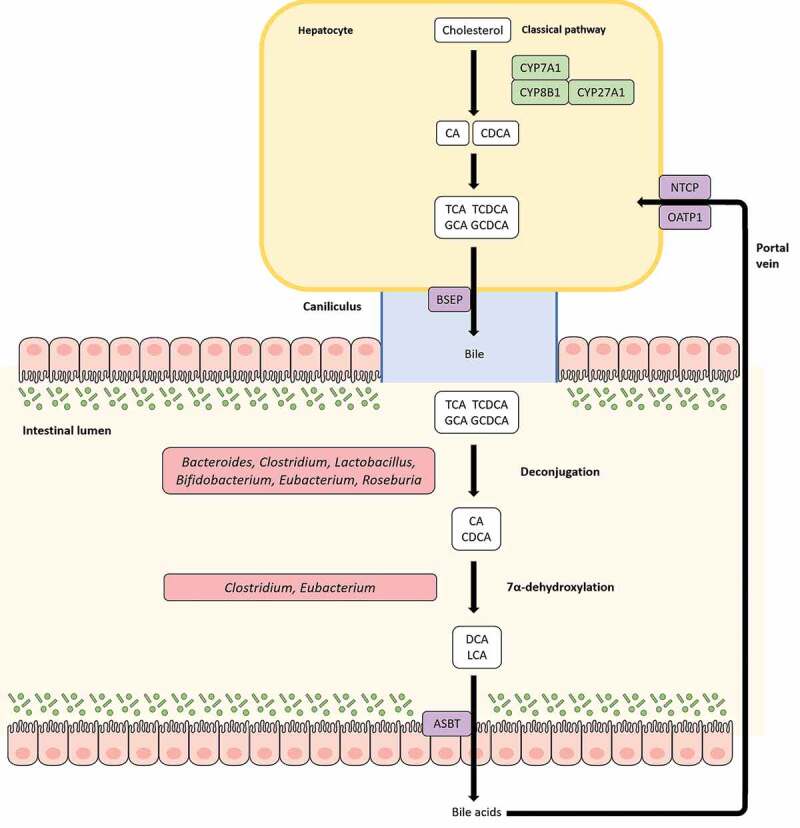
Bile acid (BA) modification by *Eubacterium* spp. and enterohepatic circulation. BAs are produced from cholesterol in the liver and are continually released into the bile canaliculi via the bile salt export pump (BSEP). The bile canaliculi drain into the gallbladder where BAs are temporarily stored and undergo postprandial release into the gut. Before release into the bile canaliculi, cholic acid (CA) and chenodeoxycholic acid (CDCA), the primary BAs produced in liver hepatocytes, can be conjugated to taurine/glycine moieties to form conjugated BAs (T/G-CA, T/G-CDCA). In the gut, primary BAs can be metabolized by gut bacteria including *Eubacterium* spp. into diverse secondary forms. BAs can undergo deconjugation to form deconjugated primary BAs and/or hydroxylation reactions to produce secondary BAs such as deoxycholic acid (DCA) and lithocholic acid (LCA). 95% of BAs are reabsorbed in the gut and recycled back to the liver through the portal vein, with conjugated BAs exhibiting highest rates of reabsorption. This circular movement of BAs from liver hepatocytes to the gut and back to the liver is known as the enterohepatic circulation.

Bile acids (BA) are host-produced metabolites derived from cholesterol in liver pericentral hepatocytes. Cholic acid (CA) and chenodeoxycholic acid (CDCA) are the primary BAs produced in liver which are then conjugated to taurine or glycine before being temporarily stored in the gallbladder; these BAs subsequently undergo postprandial secretion to reach the gut. 95% of the total BA pool in the gut are absorbed efficiently and recycled back to the liver via the portal vein; this cyclic process is known as enterohepatic circulation. The rest serves as a substrate for bacterial metabolism in the gut and constitutes a critical route for cholesterol excretion from the body. BAs can occur in several forms including primary BA, secondary BA, conjugated, or unconjugated. Various members of the gut microbiota are capable of transforming BAs, thereby influencing the composition of the local BA pool along with various other aspects of host physiology. Gut microbes including *Eubacterium* spp. that possess the enzyme bile salt hydrolase (BSH) are able to hydrolyze the C-24 N-acyl amide bond in conjugated BAs to release glycine/taurine moieties^121^ (Figure 4). Indeed, *Eubacterium* spp. along with other genera such as *Roseburia* and *Clostridium* constitute a major reservoir of BSHs in the gut.^172^ Deconjugation increases the pK_a_ of BAs to ~5, thereby making them less soluble which in turn leads to inefficient absorption and replenishment of the lost BA by *de novo* synthesis from cholesterol.^173^ Additionally, BSH activity can disrupt micelle formation and absorption, resulting in a significant reduction of cholesterol levels.^159^ Being reasonably widely distributed in the gut microbiota, BSH activity can thus be modulated to regulate weight gain and cholesterol levels in the host. Deconjugation also helps in bile detoxification through recapture and export of cotransported protons by the free BAs generated, thereby negating the pH.^174^ Another way intestinal bacteria can transform BAs is through the oxidation and epimerization of hydroxyl groups at C3, C7, and C12 positions, resulting in the generation of isobile (β-hydroxy) salts.^175^ Epimerization involves the reversible stereochemical change from α to β configuration and *vice versa*, generating a stable oxo-bile acid intermediate. This process is catalyzed by α- and β-hydroxysteroid dehydrogenases (HSDHs) and can be carried out by a single bacterial species containing both enzymes or through proto-cooperation between two species, with each contributing one enzyme. HSDH activity has been reported in several species including *Eubacterium* spp.^176^

**Figure 5.**
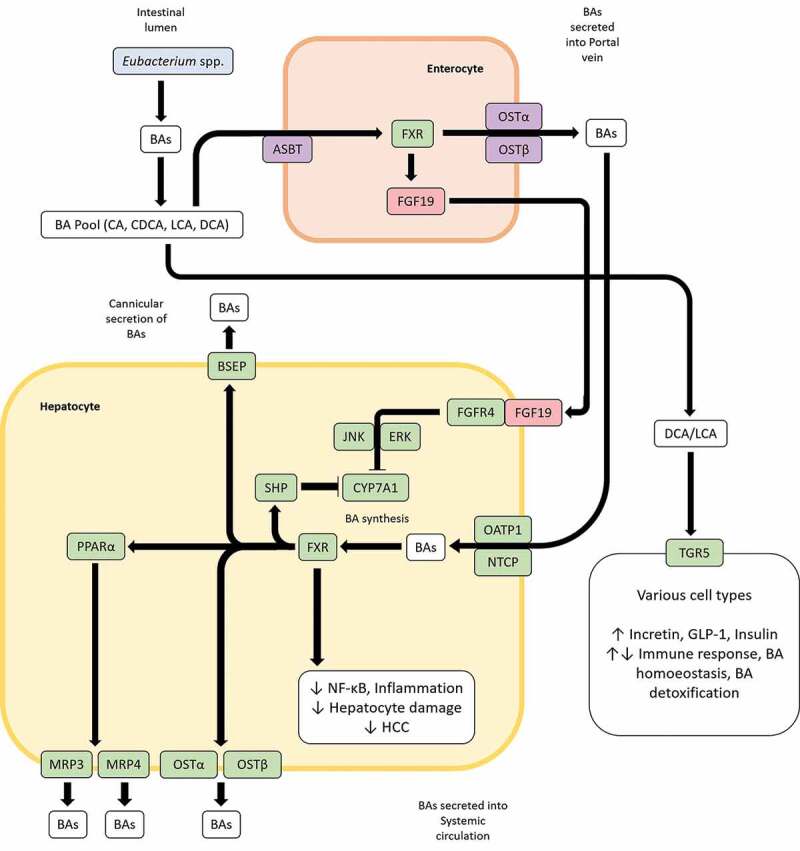
Bile acid (BA) induced signaling pathways influence BA homeostasis and inflammation. BAs in the gut are taken up by enterocytes via the apical sodium-bile acid transporter (ASBT) and bind to the farnesoid X receptor (FXR) which in turn upregulates the expression of the fibroblast growth factor 19 (FGF19). FGF19 can then bind to FGF receptor 4 in hepatocytes to downregulate BA synthesis in liver through the JNK/ERK pathway. Additionally, BAs transported through the portal vein can inhibit BA synthesis in hepatocytes in a FXR-mediated manner by entry through the organic anion transporting polypeptide 1 (OATP1) or sodium-taurocholate cotransporting polypeptide (NTCP) and upregulating the BA synthesis inhibiting transcription factor small heterodimer protein (SHP). FXR can also influence BA homeostasis through the peroxisome proliferator-activated receptor alpha (PPARα). LCA and DCA produced by *Eubacterium* spp. are high-affinity ligands for TGR5, which upon binding of said BAs can modulate glycemic response, immune response, BA homeostasis and BA detoxification in various tissues.

Bacterial 7α-dehydroxylases in the gut convert primary BAs, CA and CDCA into deoxycholic acid (DCA) and lithocholic acid (LCA), respectively (Figure 4).^173^ Although quantitatively, 7α-hydroxylation represents the most important bacterial transformation of BAs in the gut, only few distinct members of the gut microbiota such as *Eubacterium* and *Clostridium* XIVa cluster have been reported to be capable of carrying out this reaction.^173,177^ Studies on *Eubacterium* strain VPI 12708 have identified enzymes encoded by the bile acid inducible (*bai*) operon which catalyze a multistep pathway for primary BA 7α-dehyroxylation.^178^ DCA and LCA produced through 7α-dehyroxylation of primary BAs by *Eubacterium* spp. can have major impacts on gut health and homeostasis that are manifested primarily through bile acid signaling receptors. Both DCA and LCA are high-affinity ligands for the nuclear hormone receptor, farnesoid X receptor (FXR); activation of intestinal FXR by DCA or LCA upregulates the expression of the fibroblast growth factor 19 (FGF19), which in turn binds to the hepatic fibroblast growth factor receptor 4 (FGFR4) to subsequently downregulate bile acid synthesis in hepatocytes^179^ (Figure 5). FXR also promotes anti-inflammatory properties, primarily through inhibition of the NF-κB pathway and BA detoxification, through modulation of proliferator-activated receptor α (PPARα).^179^ FXR activation has also been reported to induce expression of antimicrobial peptides, thereby contributing to control of pathobionts.^173,180^ TGR5, a GPCR that activates various intracellular pathways upon interaction with BAs, also binds LCA and DCA with the highest affinity in the BA pool (Figure 5). Once activated, TGR5 stimulates the secretion of incretin hormone GLP-1 and insulin, thereby promoting energy expenditure.^181^ Additionally, TGR5 can modulate inflammatory responses, which can be both pro- or anti-inflammatory in nature; BA-TGR5 signaling plays a critical role in the intricate balance of pro- and anti-inflammatory cytokines in the gut.^179^ LCA and DCA also bind to the pregnane-X receptor (PXR), Vitamin D_3_ receptor (VDR) and constitutive androstance receptor (CAR) to variously influence BA homeostasis and BA detoxification.^179^ The strong affinity of bile acid signaling receptors for microbiota-induced secondary BAs highlight how the gut microbiota including *Eubacterium* spp. can modulate BA homeostasis, BA detoxification, control and maintenance of bacterial growth in gut, inflammation and glycemic responses through BA signaling. BA metabolism by a healthy gut microbiome also provides protection against *C. difficile* infection (CDI). DCA, which predominates in feces under healthy conditions compared to CDI subjects, can stimulate germination of C. difficile spores, but importantly, inhibits the vegetative form of *Clostridium difficile*.^182^ Dysbiosis of the gut leading to a decrease of secondary BA-producing bacteria and correlated with an increase in fecal primary BAs is permissive to the germination of *C. difficile* spores culminating in CDI.^183,184^ Indeed, restoration of gut BSH activity contributes to the efficacy of fecal microbiota transplantation (FMT) therapies in CDI patients.^185^

The gut microbiota, as modulated by diet and other factors can lead to a particular BA profile which in turn has important consequences. A high-fat diet (HFD) such as the Western diet overstimulates BA discharge into the intestine, leading to a dysbiotic gut microbiota and increased secondary BA production, especially LCA and DCA.^179^ DCA and LCA are the most hydrophobic among the BA pool and elevated levels can be cytotoxic; detrimental effects exerted by DCA and LCA can disrupt the architecture and function of the colonic epithelium through oxidative damage to DNA, triggering of pro-inflammatory responses and increased cell proliferation. In HFD-fed mice, increased LCA/DCA was correlated to an increase in the abundance of *Clostridum sordellii*, a bacterium from *Clostridium* cluster XI.^186^ Surprisingly, *Clostridium* cluster XIVa to which *Eubacterium* spp. belongs was reported as a minor contributor, even though they exhibit 7-α hydroxylation properties. Such an observation is consistent specifically for *Eubacterium* spp., which is negatively modulated by HFDs, as mentioned above. The greater reabsorption of secondary BAs in the intestine resulting from HFDs and subsequent transport to the liver causes hepatic inflammation.^179^ A reduced FXR signaling due to increased inflammation results in decreased hepatic BA transporter function and increased BA sequestration in the liver; this can establish sustained hepatic inflammation, which can eventually lead to hepatocellular carcinoma (HCC).^180^ Dysbiosis in liver disorders such as HCC, fatty acid liver disease (FLD), fibrosis and cirrhosis is additionally characterized by an elevation of aerobic, pro-inflammatory, BSH-rich bacteria such as *Enterobacter* and *Enterococcus*, which also contribute to an increased production of secondary BAs.^150^ Indeed, the ratio between primary and secondary BAs in feces and the levels of conjugated and unconjugated BAs in serum are higher in nonalcoholic FLD (NAFLD) patients.^187^ *Eubacterium* spp. is consistently found in lower proportions in liver disorders. Metagenomic shotgun sequencing of the gut microbiome of subjects suffering from fibrosis and cirrhosis has revealed a significant reduction of *Eubacterium* species such as *E. rectale, E. hallii* and *E. eligens* compared to healthy individuals^146,188-190^ (Table 3). These metagenomes also tended to be less fermentative in nature, i.e. displayed lower abundances for fermentative butyrate producers such as *Roseburia* sp., *Faecalibacterium* sp. and others, besides *Eubacterium* spp.^146, 179^

**Table 3.** Case-control studies showing association of *Eubacterium* spp. with liver diseases.

Pathology/condition/cohort description	Principal method(s) used	Inferences	Reference
86 patients with biopsy-proven NAFLD: 72 patients had stage 0–2 fibrosis and were classified as mild/moderate NAFLD and 14 patients had stage 3–4 fibrosis and were classified as advanced NAFLD.	Metagenomic shotgun sequencing	*Eubacterium rectale, Ruminococcus obeum* CAG: 39, and *R. obeum*, were significantly lower in advanced fibrosis than mild/moderate NAFLD; *E. rectale* was the most abundant organism in mild/moderate NAFLD. Indicates possible protective role of *E. rectale*.	Loomba et al.^188^
Gut metagenomic datasets from 123 patients with liver cirrhosis (LC) and 114 healthy control subjects. Metagenomes from 47 healthy individuals, 49 compensated, and 46 decompensated cirrhotic patients were finally chosen for meta-omic analysis.	*In silico* meta-omic analysis	Trends in patients with compensated and decompensated LC compared to healthy subjects: ↓ *Eubacterium rectale, Alistipes putredinis, Alistipes shahii*, and *Coprococcus eutactus* ↑ *Haemophilus parainfuenzae, Streptococcus* *salivarius, Lactobacillus salivarius*, and *Veillonella parvula*	Shao et al.^189^
Gut metagenomes from 98 Chinese LC patients and 83 healthy volunteers.	Metagenomic shotgun sequencing	*Veillonella, Streptococcus, Clostridium and Prevotella* were enriched in the liver cirrhosis group, while *Eubacterium* and *Alistipes* were dominant in the healthy controls.	Qin et al.^190^

Secondary bile acids as produced by the gut microbiota may also play a critical role in the development and establishment of CRC. As mentioned above, butyrate inhibits colorectal carcinogenesis and a marked reduction of butyrate producers in the gut including *Eubacterium* spp. is commonly observed in patients with CRC. Several butyrate producers including *Eubacterium* spp., which belong to *Clostridium* cluster XIVa can additionally produce secondary BAs through 7-α hydroxylation of primary BAs.^173^ In IBD subjects with chronic inflammation of the gut, significantly lower levels of secondary BAs with concurrently increased fecal-conjugated BAs and a marked decrease of *Clostridium* cluster XIVa is reported.^87,101,173,191^ A reduction in secondary BA levels contribute to a loss of the anti-inflammatory effects of secondary BAs on intestinal epithelial cells, thereby enhancing the chronic inflammation.^191^ Even though the loss of butyrate producers and secondary BA producers in IBD have been made separately, the two groups share significant overlap and both are depleted in chronic inflammation of the gut.^87,101,150,173,191^ Indeed, a recent bioinformatic analysis of gut metagenomes has revealed significantly decreased populations of butyrate producers *F. prausnitzii* and *E. rectale* in IBD patients.^192^ Understandably, an absence of this group of butyrate and secondary bile acid-producing bacteria that includes *Eubacterium* spp. promotes the development of IBD and its eventual progression to CRC, where a similar dysbiotic gut microbiome is observed.^136,152,153^ Indeed, modulation of bile acid profiles and/or gut microbiota are being pursued as novel therapeutic approaches for HCC and CRC.

## *Eubacterium* spp. are involved in critical metabolic transformations in the gut

Metabolic transformations of specific compounds in the gut by the resident microbiota can be critical to human health. Substances can be taken up in the intestine that cannot be detoxified or broken down by the human body and thus, can result in toxicogenic effects. *Eubacterium* spp. have been shown to be capable of carrying out important metabolic transformations in the gut with positive effects on human health including detoxification of toxic compounds into much more benign forms. Multiple beneficial transformations by *E. hallii* were recently reported by Fekry et al. In their study, Fekry et al. found *E. hallii* to be highly proficient in the transformation of a highly abundant food-derived heterocyclic aromatic amine carcinogen – 2-amino-1-methyl-6-phenylimidazo(4,5-*b*)pyridine (PhIP) into a biologically unavailable form – 7-hydroxy-5-methyl-3-phenyl-6,7,8,9-tetrahydropyrido[3′,2′:4,5]imidazo [1,2-α]pyrimidin-5-ium chloride (PhIP-M1).^193^ Additionally, PhIP transformations by *E. hallii* in the presence of simulated proximal and distal colon microbiota led to a 300-fold and 120-fold increase in its abundance, respectively, indicating great potential for use as a protective agent. In the same study, Fekry et al. also observed *E. hallii* to be capable of metabolizing glycerol to 3-hydroxypropionaldehyde (3-HPA), which exists as reuterin in aqueous solutions. Interestingly, reuterin has been shown to have inhibitory effects against Gram-positive and Gram-negative bacteria, fungi and yeast, possibly through increasing oxidative stress by modulating intracellular glutathione, thereby making it an attractive target for therapeutics.^194^

In another instance, the transformation of 8-prenylanringenin (8-PN) from isoxanthohumol (IX) by gut microbes was investigated by Possemiers et al.^195^ 8-PN is known as a potent phytoestrogen and has been used to alleviate symptoms of menopause.^196^ Production of 8-PN from IX, found commonly in hops and beers, has been found to be highly variable between individuals.^197^ In their study, Possemiers et al. carried out supplementation of *E. limosum*, a bacterium known to carry out the transformation from IX to 8-PN and found that germ-free rats could indeed be induced by *E. limosum* to produce greater levels of 8-PN from IX upon transplantation of the microbiota from low 8-PN producing individuals. This probiotic effect of *E. limosum* requires further investigation if it can be applied to humans, especially with respect to the potential for variations in duration of effect and between individuals in terms of colonization efficiencies and other factors. The metabolic transformations described above add greatly to our understanding of the diverse array of benefits humans derive from gut *Eubacterium* spp. besides production of SCFAs. However, as highlighted already, further research is necessary to truly exploit all the potential benefits the *Eubacterium* genus has to offer.

## Conclusion

The genus *Eubacterium* is a phylogenetically diverse group of microbes, a fact that makes associated taxonomic assignments challenging. Regardless, many current and former members of this genus exhibit compelling associations with gut health, and, as a major butyrate producer and core gut microbiota component, are immensely important. In this review, we have discussed how *Eubacterium* spp. is involved in various aspects of gut health through important contributions in SCFA, cholesterol and bile acid metabolism in the gut; we have also elaborated the phylogenetic characteristics of the genus and how it is modulated in the gut by diet and age. In the process we have outline how *Eubacterium* spp. play a major role in modulation of inflammation, regulation of immune responses, maintenance of barrier integrity in the gut, moderating glycemic response, and cholesterol homeostasis, among others. Strong correlations with beneficial effects in several clinical conditions have prompted further interest in the genus, with multiple species being considered for commercial endeavors as next generation probiotics/biotherapeutics.^154,198-201^ Most notably, efforts are underway at Caelus Health, in collaboration with Danish bioscience firm Chr. Hansen, to create oral formulations containing *E. hallii* strains as a biotherapeutic to reduce insulin resistance in individuals with metabolic syndromes and to prevent the development of T2DM.^198^ Given that the gut is a highly competitive and functionally non-redundant environment, recurring associations of *Eubacterium* spp. with positive clinical phenotypes combined with a simultaneous resolution of its modes of action establishes a consensus on its positive influence on human health. However, further studies are required to attribute causality to observed associations, i.e., understanding pathogenesis of clinical conditions with respect to gut microbiota. In what remains a major caveat in gut microbiology research today, our understanding of how much the gut microbiota – across all relevant species – influences a clinical condition and vice versa is still limited. Longitudinal studies with tightly controlled diet regimens where the gut microbiota and relevant health parameters are evaluated over protracted time periods may be necessary to elaborate such causalities. Even then, attribution of causation to specific species may prove to be difficult due to the tightly clustered functional niches in the gut.

A recurring motif suggests that in several clinical conditions, especially metabolic syndromes, diet, lifestyle, and other factors can induce the dysbiosis of the gut microbiota, which in turn creates an undesirable metabolic profile. The change in the effective proportions of these metabolites which, directly or indirectly modulate inflammation, barrier integrity, energy homeostasis, and so on, plays an important role in the development and progression of disease pathogenesis. Given the complexity of the processes involved, host-metabolite-microbiota crosstalk must be approached from a system biology standpoint using technologies such as metagenomics and metabolomics. It may be necessary to study involved components together and not in isolation, with therapeutic solutions aimed at modulation of all these components. To this end, further *in vitro* and *in vivo* characterization of *Eubacterium* spp. at the genomic, metagenomic and eventually at the ecological level is required. This will allow us to better understand how the relatively understudied *Eubacterium* spp. interacts with other members of the gut microbiome and how they are modulated by host factors and diet. Garnering such an understanding is crucial to the successful control and prevention of clinical conditions using clusters of commensal bacteria producing critical metabolites, as evidenced by Geirnhaert et al.^101^ Much remains to be understood about the metabolic activities, immunomodulatory influences, and ecological role of *Eubacterium* spp., both in isolation and in combination, with other potential next-generation health-promoting microorganisms such as *Akkermansia muciniphila* and *F. prausnitzii*, to ensure its effective deployment in evidence-based gut therapeutics. However, based on evidence to date, there is a lot of cause for optimism.
